# Ketamine and selective activation of parvalbumin interneurons inhibit stress-induced dendritic spine elimination

**DOI:** 10.1038/s41398-018-0321-5

**Published:** 2018-12-10

**Authors:** Lhotse Hei Lui Ng, Yuhua Huang, Lei Han, Raymond Chuen-Chung Chang, Ying Shing Chan, Cora Sau Wan Lai

**Affiliations:** 1 School of Biomedical Sciences, The University of Hong Kong, Pokfulam, Hong Kong SAR China; 2 State Key Laboratory of Cognitive and Brain Research, The University of Hong Kong, Pokfulam, Hong Kong SAR China

## Abstract

Stress is a major risk factor for the onset of many psychiatric diseases. In rodent models, chronic stress induces depression and impairs excitatory neurotransmission. However, little is known about the effect of stress on synaptic circuitry during the development of behavioral symptoms. Using two-photon transcranial imaging, we studied the effect of repeated restraint stress on dendritic spine plasticity in the frontal cortex in vivo. We found that restraint stress induced dendritic spine loss by decreasing the rate of spine formation and increasing the rate of spine elimination. The *N*-methyl-d-aspartate receptor antagonist ketamine inhibited stress-induced spine loss mainly by protecting mushroom spines from elimination. Ketamine also induced re-formation of spines in close proximity to previously stress-eliminated spines. Electrophysiological and in vivo imaging experiments showed that ketamine enhanced activity of parvalbumin (PV) interneurons under stress and counterbalanced the stress-induced net loss of PV axonal boutons. In addition, selective chemogenetic excitation of PV interneurons mimicked the protective effects of ketamine on dendritic spines against stress. Collectively, our data provide new insights on the effects of ketamine on synaptic circuitry under stress and a possible mechanism to counteract stress-induced synaptic impairments through PV interneuron activation.

## Introduction

Dendritic spines are small protrusions on dendrites, where the postsynaptic part of most excitatory synapses is located. They are highly dynamic structures in terms of number and morphology^1–3^. The structural and physiological properties of dendritic spines are crucial for local signal integration, signal transduction, and molecular compartmentalization^4–8^. Substantial changes in spine number and morphology have been associated with neurological and psychiatric diseases such as Alzheimer’s disease, epilepsy, schizophrenia, and autism spectrum disorders (ASD); reflecting the vital role of dendritic spines for the functioning of central nervous system^9,10^.

Stress is a risk factor for many psychiatric diseases including major depressive disorder and post-traumatic stress disorder^11,12^. Animal models of depression induced by chronic stress showed deficits in excitatory transmission as well as structural changes in excitatory neurons in the frontal cortex, including dendritic branch atrophy and dendritic spine loss^13–16^. These functional and structural changes echo with the hypofunction and the decrease in volume of frontal cortex in major depressive disorder patients^17–21^. These findings suggest that structural alterations at the synaptic level provide a substrate for the pathogenesis of depression. Nonetheless, most studies were performed using post-mortem tissues at fixed time points, which are unable to provide a full picture regarding the pathological changes of the highly dynamic dendritic spines during depression development.

Ketamine is a non-competitive *N*-methyl-d-aspartate (NMDA) receptor antagonist that is commonly used for anesthesia^22,23^. Recent studies show that a single sub-anesthetic dose of ketamine exerts robust antidepressant effects on treatment-resistant patients within hours after administration, which persists for several days to up to a week^24,25^. This has drawn attention to the potential use of ketamine as a treatment for depression. Animal studies reveal that ketamine rapidly induces antidepressant-like effects and increases dendritic spine density^26–30^. Nevertheless, it is unclear how ketamine affect dendritic spine dynamics under stressed condition in vivo.

Ketamine has been proposed to enhance excitatory drive through preferential inhibition of gamma-aminobutyric acid (GABA)-ergic interneurons^31–33^. GABAergic interneurons account for 20–30% of neurons in the cerebral cortex and play a crucial role in neural circuitry and activity by synchronizing and orchestrating pyramidal neuron firing^34–36^. Deficits in inhibitory neurotransmission have been implicated in psychiatric disorders such as schizophrenia, ASD, and depression^37–39^. In the cortex, there are three main subtypes of interneurons that express parvalbumin (PV), somatostatin (SST), and vasointestinal peptide (VIP) in a non-overlapping manner^35,40^. PV interneurons are the largest subset of interneurons in the cortex, which are fast-spiking and exert powerful inhibition to pyramidal neurons^40–44^. Although ketamine at a high, psychotic dose was found to reduce the number of PV interneurons in the prefrontal cortex^45,46^, it is unknown how ketamine at antidepressant dose would affect PV interneurons under stress. Interestingly, a recent study showed that selective activation of PV interneurons ameliorated stress-induced learning deficits in barrel cortex^47^. However, it is unclear if the effects of ketamine involve the activation or inhibition of PV interneurons in the frontal cortex under stress.

In this study, we investigated the effect of ketamine on dynamic change of dendritic spines in repeatedly stressed mice by in vivo two-photon transcranial imaging. We found that ketamine at antidepressant dose prevented stress-induced dendritic spine elimination. Ketamine also increased PV interneuron activity and prevented net loss of PV axonal boutons under stressed condition. Moreover, selective chemogenetic activation of PV interneurons mimicked, whereas inhibition of PV interneurons abolished the protective effects of ketamine on dendritic spines. Altogether, our data suggest that activation of PV interneurons plays a role in the modulation of dendritic spine plasticity by ketamine in response to stress.

## Materials and methods

### Animals

Mice were group-housed under a 12 h light/dark cycle, with food and water available ad libitum. *Thy1-*YFPH, *PV-*Cre, *SST*-Cre, *VIP*-Cre, and Ai9-tdTomato mice with C57BL/6 background were purchased from the Jackson Laboratory and housed in the Laboratory Animal Unit, The University of Hong Kong. Postnatal day 30 (P30) ± 1 mice were used in this study. Animals were randomly assigned to different experimental groups. All experiments were approved and performed in accordance with University of Hong Kong Committee on the Use of Live Animals in Teaching and Research guidelines.

### Restraint stress (RS)

This stressor consisted of placing each mouse in a well-ventilated 50-ml transparent tube for 6 h per day (1000–1600). Mice were returned to their home cage and had access to food and water ad libitum after each restraint period. The non-stressed control mice were left undisturbed in their home cages.

### Drugs application

Mice were administered with ketamine at a dose of 10 mg/kg by intraperitoneal injection before each RS session. For the chemogenetic experiment, clozapine N-oxide (CNO) (Sigma-Aldrich) was injected at a dose of 10 mg/kg intraperitoneally, before each RS session.

### In vivo imaging of dendritic spines and PV interneuron axonal boutons

For dendritic spine imaging, spine formation, and elimination were examined longitudinally by imaging the mouse cortex through a thinned skull window as described previously^48,49^. In brief, *Thy1-*YFPH mice expressing YFP in layer five (L5) pyramidal neurons at P30 ±1 day were imaged. Mice were head fixed and thinned skull windows were made with high-speed microdrills. Skull thickness was reduced to ~ 20 µm. For re-imaging of the same region, previously thinned regions were identified based on the maps of the brain vasculature. Microsurgical blades were used to re-thin the region of interest (ROI) until a clear image could be obtained. As repeated thinning of the skull to ~ 20 µm without damaging the cortex becomes more difficult after multiple imaging sessions, we designed our experiments in such a way that no animal was imaged more than three times. For PV axonal bouton imaging, a cranial window of 2 mm × 2 mm was implanted over the frontal association cortex 10 days prior to imaging at P30 ± 1 as described previously^50^. Axons of PV interneurons from L2/3 cortex of *PV*-Cre/ Ai9-tdTomato mice were imaged. A two-photon microscope (Olympus FVMPE-RS) tuned to 920 nm for spine imaging and 1080 nm for PV bouton imaging (25× water immersion lens, N.A. = 1.05) was used to acquire images. For both spine and PV bouton imaging, the area of the imaging region is 216 µm × 216 µm and the center of imaging region is located at the frontal association cortex ( + 2.8 mm bregma, + 1.0 mm midline) and all imaged mice were anesthetized with ketamine/xylazine (i.p., 20 mg/ml and 3 mg/ml, respectively, in saline, 5 µl/g body weight)^51^.

### Adeno-associated viral (**AAV**) vector injection

Cre-dependent AAV-hSyn-DIO-hM4D(Gi)-mCherry or AAV-hSyn-DIO-hM3D(Gq)-mCherry was used to drive hM4D or hM3D expression in specific interneurons in *PV*-Cre/*Thy1*-YFPH, *SST*-Cre/*Thy1*-YFPH and *VIP*-Cre/*Thy1*-YFPH mice (AAV, serotype 5; hM3D: 6.4 × 10^12^ viral particles per ml, hM4D: 6.5 × 10^12^ viral particles per ml; produced by University of North Carolina Vector Core). Cre-dependent AAV-Syn-Flex-GCaMP6f was used to express the genetically encoded calcium indicator GCaMP6f specifically in PV interneurons of *PV*-Cre/Ai9-tdTomato mice (AAV, serotype 5; viral particles 1.82 × 10^13^ per ml; produced by University of Pennsylvania Vector Core). AAV virus is diluted with artificial cerebrospinal fluid (ACSF) at a 1:2 ratio and slowly injected by Picopump (10 p.s.i., 0.8 Hz) over 10–15 min into the frontal association cortex bilaterally and waited 10–14 days for viral expression.

### In vivo calcium imaging of PV interneurons

One day prior to imaging, a head holder was mounted onto the skull of the mouse to restrain its movement during imaging as described previously^50^. A glass cranial window was then implanted over the frontal association cortex to allow for imaging of GCaMP6f in PV interneurons from L2/3 at the depth of 200–300 μm below pial surface. During imaging, awake mice were restrained onto a metal plate via the head holder (Fig. 4d), and the mice were kept restrained for 8 h. GCaMP6f signal was collected at 4 and 8 h after RS. A two-photon microscope tuned to 920 nm (25× water immersion lens, N.A. = 1.05) was used to acquire images. At each time point, images (512 × 512 pixels) covering an area of 509 × 509 μm were taken over a 2-min session at 2 Hz from the same ROI. Saline or ketamine was administrated right after the imaging session at 4 h.

### Data analysis

All data analysis was performed blind to treatment conditions. For imaging of dendritic spines, dendritic branches were randomly sampled within a 216 µm × 216 µm area imaged at 0–100 µm distance below the pia surface. The same dendritic segments were identified from three-dimensional image stacks taken at different time points with high image quality (ratio of signal to background noise > 4:1). The number and location of dendritic protrusions (protrusion lengths were more than one-third of the dendritic shaft diameter) were identified. Filopodia were identified as long, thin structures (generally larger than twice the average spine length, ratio of head diameter to neck diameter < 1.2:1 and ratio of length to neck diameter > 3:1). The remaining protrusions were classified as spines^48,49^. For study of spine morphology, stubby spines were defined as spines without necks. Spines with necks were either classified as mushroom spines, which have head width > 0.6 µm and head to neck ratio > 1.5, or thin spines with head width < 0.6 µm or/and head to neck ratio < 1.5^52,53^. Three-dimensional image stacks were used for analysis to ensure that tissue movements and rotation between imaging intervals did not hinder spine identification. Spines or filopodia were considered as identical between views if their positions were unchanged with respect to adjacent landmarks. For imaging of PV axonal boutons, axonal branches were randomly sampled within a 216 µm × 216 µm area imaged at the depth at 0–300 μm below pia surface. The same axon segments were identified from three-dimensional stacks taken at different time points with high image quality (ratio of signal to background noise > 4:1). The number and location of axonal boutons, i.e., swellings along axonal shaft that are two times brighter than the shaft, were identified^54^.

The percentage of spine/bouton formation and elimination represented the number of spines/ boutons formed or eliminated between the first and second view divided by the total number of spine/bouton counted at the first view in each individual mouse. Net change in number of spine/bouton was presented as the rate of spine/bouton formation minus the rate of spine/bouton elimination. The percentage of spine elimination of different spine subtypes was presented as number of mushroom, stubby or thin spines eliminated divided by total number of spines counted at the first view. Unless specified, the *n* number for analysis of imaging experiments refers to the number of animals analyzed in each group. For imaging experiments, sample size is determined based on previous studies that performed similar types of experiment^49–51,54^. For dendrite and axon image display, fluorescent structures near and out of the focal plane of the dendrites or axons of interest were removed manually from image stacks using Adobe Photoshop. The modified image stacks were then projected to generate two-dimensional images and adjusted for contrast and brightness. Tables showing the number of dendritic spine/PV bouton analyzed, the number and gender of animal used are included in the supplementary information (Supplementary table 1 and 2).

For calcium imaging, image J software was used for analysis. Visually identifiable soma with a signal to noise ratio > 2: 1 were subjected to analysis. ROI was drawn manually around soma and measured for signal intensity. Mean signal intensity (*F*) of each ROI was used for analysis. Change in fluorescence signal (ΔF/F_0_) was calculated as (F at different time points−baseline *F*)/baseline *F*. The average reading from the first 10-s period of the 2-min imaging session was used as the baseline *F*_0_. Reading at 4 h was used as *F*_0_ for studying the effect of ketamine on PV interneuron activity under stressed condition.

### Whole-cell patch-clamp recordings

*PV*-Cre/Ai9-tdTomato mice were stressed for 7 days from P30–37. One day after the last session of stress, brain sections containing the frontal cortex were prepared as previously described^55^. Mice were decapitated quickly after isoflurane inhalation and brains were taken out from the skull. The brains were then removed to ice-cold and oxygenated ACSF containing (in mM) 120 NaCl, 2 KCl, 2.5 CaCl_2_, 1.2 MgCl_2_, 1.2 KH_2_PO_4_, 26 NaHCO_3_, and 11 glucose. This extracellular solution had 302–310 mOsm in osmolality and bubbled with 95% O_2_ and 5% CO_2_ to reach a pH value ~ 7.3. Sagittal sections containing the frontal cortex were cut at a thickness of 300 μm with a vibratome (Campden Instruments, UK). The freshly obtained brain slices were then immediately transferred to a pre-warmed (33 °C) water bath incubator (Grant Instruments Ltd, UK) and kept for at least 1 h in oxygenated ACSF before patch-clamp recording. After incubation, slices were transferred to a recording chamber with oxygenated ACSF (2.0–2.5 ml/min) at room temperature. The slices were placed on an upright infrared video microscope with differential interference contrast optics (Olympus BX51WI, Japan). Neurons in the frontal association cortex were visualized through a × 40water immersion objective lens using an infrared-sensitive camera (Sony, Japan). Whole-cell patch-clamp recordings were conducted with glass pipettes (Harvard Apparatus, USA) of resistance 4–6 MΩ pulled by a horizontal micropipette puller (Model P-97, Sutter Instruments, Novato, CA, USA) and backfilled with internal solution consisting of following components (in mM): 130 Kgluconate, 8 KCl, 2 MgCl_2_, 2 NaCl, 1 EGTA, 10HEPES, 2 Na_2_ATP, 0.3 Na_3_GTP (pH value was adjusted to 7.3 with KOH, 290 mOsm). Action potentials were recorded by holding the membrane potential at − 70 mV and injecting 1 s step currents to elicit action potentials. Signals were recorded using an Axon MultiClamp 700B amplifier controlled by Clampex 10.2 software (Molecular Devices, Sunnyvale, CA, USA). Signals were filtered at 3 kHz and digitized at 10 kHz. Recording data were employed only when series resistance was lower than 20 MΩ and did not change by > 20%. PV interneurons from all cortical layers in frontal association cortex (FrA) were picked randomly for recording.

### Statistical analysis

All statistical analyses were performed using GraphPad Prism software 7.0 (GraphPad Software, La Jolla, CA, USA). The Shapiro–Wilk test was used to test for normality of all data sets. For comparison between all data sets with normal distribution, Student’s *t* test or one-way ANOVA followed by post hoc analyses using Tukey’s test was used. For Student’s *t* test, *F* test was used to test for homogeneity of sample variances, and Welch’s correction is used when samples do not have equal variances. For comparison including one or more data sets that does not follow normal distribution, Mann–Whitney *U* test or Kruskal–Wallis test followed by post hoc Dunn’s test was used. In all analyses, *P* values < 0.05 were considered as statistically significant.

## Results

### Repeated RS induces dendritic spine loss by the promotion of spine elimination and the inhibition of spine formation

**Fig. 1:**
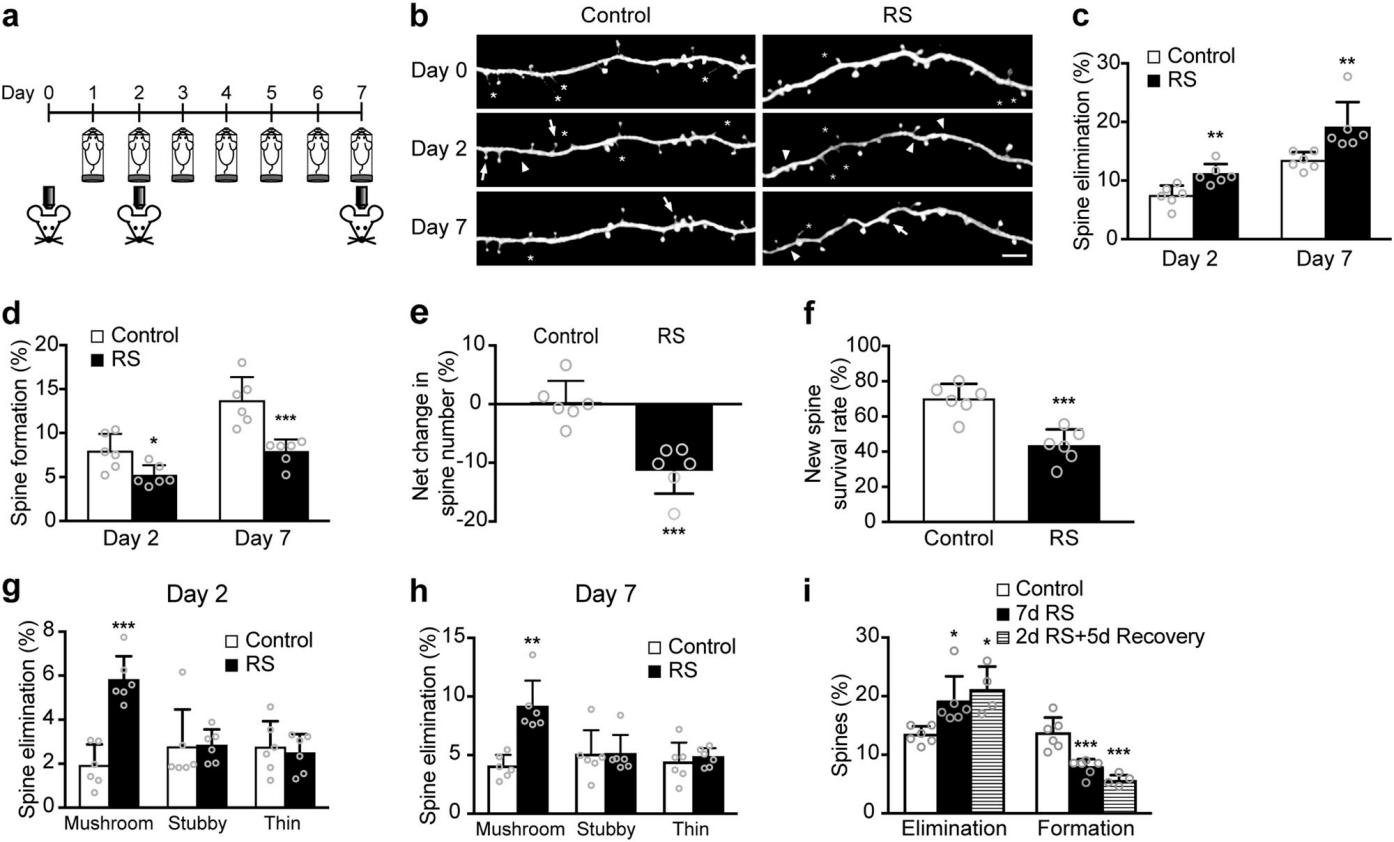
Effects of repeated restraint stress on dendritic spine plasticity in the frontal association cortex (FrA). **a** Timeline of restraint stress (RS) treatment and imaging. **b** Representative image of dendritic branches in the FrA from control and RS groups. Arrowheads mark sites of spine elimination. Arrows mark sites of newly formed spines. Asterisks mark filopodia. Scale bar 4 µm. **c**, **d** Rate of dendritic spine elimination and formation in control (*n* = 6, 929 spines) and RS groups (*n* = 6, 910 spines). **e** Net change in dendritic spine number in control and RS groups at day 7. **f** Percentage of newly formed spines at day 2 that survived at day 7. **g**, **h** Percentage of eliminated spines classified as mushroom, stubby, and thin spines after 2 and 7 days of RS. *P < 0.05, **P < 0.01, ***P < 0.001 compared with control, Student’s *t* test **c**–**h** except Mann–Whitney *U* test for 7-day elimination in **c** and mushroom spine elimination in **h**. **i** Rate of dendritic spine elimination and formation in control, 7d RS and 2d RS + 5d Recovery (*n* = 4, 504 spines) groups. Elimination: *P* = 0.0004, *H* = 10.88, Kruskal–Wallis test. Formation: *P* < 0.0001, *F*_(2, 13)_ = 23.39, one-way ANOVA. *P < 0.05 compared with RS. Data are presented in mean ± s.d

To understand the short- and long-term effects of stress on dendritic spine dynamics, the mouse frontal association cortex (FrA) was studied longitudinally by *in vivo* transcranial imaging in *Thy1*-YFP transgenic mice that express yellow fluorescent protein in layer V (L5) pyramidal neurons. The FrA is readily accessible by non-invasive transcranial imaging and is connected with other brain regions such as the medial prefrontal cortex and amygdala in rodents^51,56,57^, which have been shown to be involved in stress response and affected in depression^58^. Mice were imaged at day 0, 2 and 7 under daily RS (Fig. 1a, b). We found that two days of RS significantly increased the rate of spine elimination (*P* = 0.0045, Fig. 1c) and decreased the rate of spine formation when compared with control (*P* = 0.0179, Fig. 1d). The increase in spine elimination (*P* = 0.0022) and decrease in spine formation (*P* = 0.0010) persisted after 7 days of stress (Fig. 1c, d). The combination of enhanced spine elimination and reduced spine formation under stress led to a significant net loss of spines after 7 days of RS (*P* = 0.0005, Fig. 1e). In addition, RS significantly decreased the survival rate of newly formed spines when compared with control at day 7 (*P* = 0.0006, Fig. 1f). Morphological analysis of the eliminated spines showed that the additional spines eliminated under RS were mostly mushroom spines (Day 2, *P* < 0.0001; day 7, *P* = 0.0022, Fig. 1g, h), which is considered to be the more mature and stable spine type in neural circuits^2^; whereas the rate of elimination for both stubby and thin spines were similar in control and RS groups.

To examine if dendritic spine deficits induced by short-term stress can be restored by a stress-free recovery period, mice were stressed for 2 days followed by a 5-day recovery period (2d RS + 5d recovery). We found that mice still showed a significantly higher spine elimination rate (*P* = 0.0102) and a lower spine formation rate (*P* < 0.0001) after 5 days of stress-free recovery when compared to control group (Fig. 1i). In short, these data show that short-term stress (2 days) can already exert drastic effects on dendritic spine dynamics as reflected by a significant elimination of mature synaptic connections and reduction in spine formation, which cannot be reverted by stress-free recovery.

### Ketamine ameliorates the loss of dendritic spines by protecting mushroom spines from elimination under repeated RS

**Fig. 2:**
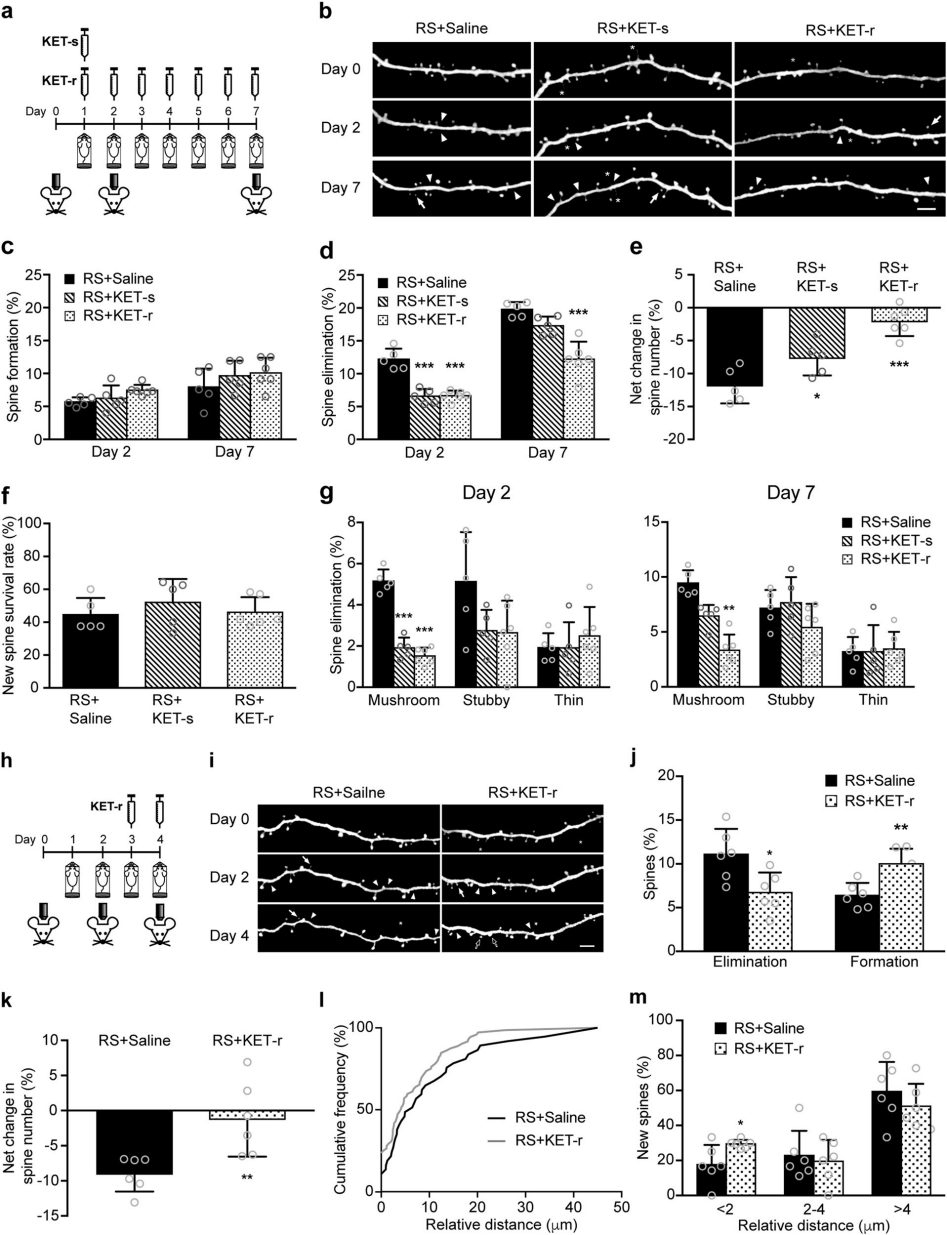
Ketamine counteracts RS-induced elimination of mature dendritic spines. **a** Timeline of ketamine treatment and imaging under RS. In single dose ketamine (KET-s) group, ketamine was administered on day 1 before RS session. In the repeated ketamine (KET-r) group, ketamine was injected daily before RS session. **b** Representative image of dendritic branches in the FrA from saline, KET-s, and KET-r groups under RS. Arrowheads mark sites of spine elimination. Arrows mark sites of newly formed spines. Asterisks mark filopodia. Scale bar 4 µm. **c**, **d** Rate of dendritic spine formation and elimination in RS + saline (*n* = 5, 774 spines), RS + KET-s (*n* = 5, 728 spines), and RS + KET-r groups (*n* = 6, 892 spines). Formation: Day 2: *P* = 0.0908, *F*_(2, 13)_ = 2.901; day 7: *P* = 0.3535, *F*_(2, 13)_ = 1.128, one-way ANOVA. Elimination: Day 2, *P* < 0.0001, *F*_(2,13)_ = 38.78, one-way ANOVA. Day 7, *P* < 0.0001, *F*_(2,13)_ = 22.31, one-way ANOVA. **e** Net change in dendritic spine number in RS + saline, RS + KET-s, and RS + KET-r groups at day 7. *P* < 0.0001, *F*_(2,13)_ = 21.22, one-way ANOVA. **f** Percentage of newly formed spines at day 2 that survived at day 7 in RS + saline, RS + KET-s, and RS + KET-r groups, *P* = 0.5496, *F*_(2,13)_ = 0.627, one-way ANOVA. **g** Percentage of eliminated spines classified as mushroom, stubby, and thin spines after 2 and 7 days of RS. Mushroom spines: Day 2, *P* < 0.0001, *F*_(2,13)_ = 87.99, one-way ANOVA. Day 7, *P* < 0.0001, *H* = 12.88, Kruskal–Wallis test. **P < 0.01, ***P < 0.001 compared with RS + saline. Data are presented in mean ± s.d. **h** Timeline of ketamine treatment and imaging under pre-stressed condition. **i** Representative image of dendritic branches in the FrA of saline and KET-r groups under RS. Arrowheads mark sites of spine elimination. White arrows mark sites of newly formed spines. Asterisks mark filopodia. Hollow arrows indicate formation of new spines at day 4 within 2 µm of spines eliminated at day 2. Scale bar 4 µm. **j** Rate of dendritic spine elimination and formation in RS + saline (*n* = 6, 735 spines) and RS + KET-r groups (*n* = 6, 830 spines) from day 2 to day 4. **k** Net change in dendritic spine number in RS + saline and RS + KET-r groups at day 4. **l** Cumulative frequency plot showing the relative distance between new spines formed at day 4 and the nearest spine that was eliminated at day 2. **m** Graph showing the relative distance of spine newly formed at day 4 to the nearest spine eliminated at day 2. RS + saline: 37 spines, RS + KET-r: 72 spines. *P < 0.05, **P < 0.01 compared with RS + saline. Student’s *t* test. Data are presented in mean ± s.d

As single, sub-anesthetic dose of ketamine has been found to increase dendritic spine density in the frontal cortex of both naive and chronically stressed rodent^26,27,30^, we studied if ketamine could modulate stress-induced deficits in dendritic spine dynamics (Fig. 2a, b). We first examined the effect of single dose ketamine (KET-s) on dendritic spine dynamics in RS mice. The KET-s group (*n* = 5) showed no significant difference in spine formation rate, but the spine elimination rate was significantly lower at day 2 when compared with saline control (*n* = 5) (*P* < 0.0001, Fig. 2c, d). However, the effect on spine elimination was transient and disappeared at day 7 (*P* = 0.1410, Fig. 2d). To understand if the transient effect of single dose ketamine was owing to its transient nature or reaching a plateau, mice were injected with ketamine daily before the start of RS session for 7 days. Repeated doses of ketamine (KET-r) (*n* = 6) significantly and persistently inhibited RS-induced spine elimination (Day 2 and day 7, both *P* < 0.0001, Fig. 2d). KET-r induced a notable increase in spine formation in stressed mice at day 2 (Fig. 2c), but the effect was transient. In addition, we found that KET-r increased spine formation rate at day 2 in non-stressed mice (*P* = 0.0465, Supplementary Figure 1), but had no effects on spine elimination rate, suggesting the effect of KET-r on spine elimination is only prominent under stressed condition. Overall, both KET-s and KET-r significantly reduced the net loss of spines under RS (*P* = 0.0480 and *P* < 0.0001, respectively, Fig. 2e). Stressed mice treated with KET-r showed a similar net change in spine number, whereas KET-s showed a significantly higher net spine loss when compared with non-stressed control (Supplementary Figure 2a–c). Nonetheless, ketamine did not alleviate RS-induced decrease in the survival of newly formed spines (Fig. 2f). Next, we further examined the morphology of spines that were eliminated after 2 days of stress. We found that ketamine significantly reduced the elimination of mushroom spines particularly (Day 2: *P* < 0.0001 for both KET-s and KET-r, day 7: *P* = 0.0010 for KET-r), but had no effect on stubby or thin spines (Fig. 2g). In short, ketamine ameliorated RS-induced spine loss through protecting mushroom spines from stress-induced elimination instead of promoting spine formation. Moreover, the effect of ketamine was transient and repeated doses were required to counteract stress-induced spine loss. In addition, since the ketamine/xylazine mixture used for anesthesia, we also examined the effect of ketamine when using isoflurane as anesthetics. We found that under isoflurane anesthesia, repeated ketamine treatment still significant reduced the rate of spine elimination in stressed mice (Day 2: *P* = 0.0480 and day 7: *P* = 0.0286). There was also no effect on spine formation rate compared with saline control (Supplementary Figure 3a, b). These data showed that the observed effects of ketamine against stress were not confounded with the anesthesia.

### Ketamine induces formation of spines in close proximity to previously stress-eliminated spines

As short-term stress (2 days) already exerted drastic effects on dendritic spine dynamics, we next examined if ketamine could exert a protective effect on spines after pre-exposure to stress. Mice were first pre-stressed for 2 days and then given saline or repeated doses of ketamine while subjected to RS for another 2 days (Fig. 2h, i). Even after pre-exposure to stress, KET-r still significantly inhibited spine elimination (*P* = 0.0161) and promoted spine formation in RS mice over two days of subsequent stress (*P* = 0.0027, Fig. 2j). This resulted in a significant reduction in net spine loss in KET-r group compared with saline group over the 4 days of stress (*P* = 0.0087, Fig. 2k). As ketamine transiently promoted formation of spines, we also examined the location of newly formed spines to see if ketamine would promote re-formation of the spines lost under previous stress exposure. The relative distance between the spines newly formed after ketamine treatment and the previously eliminated spine was measured. There was a higher percentage of new spines formed within a shorter distance to previously eliminated spines after ketamine treatment compared with saline group (Fig. 2l). Furthermore, ketamine promoted the formation of spines in close proximity (≤ 2 µm) to the location of previously eliminated spines (*P* = 0.0488, Fig. 2m). These data suggest that ketamine may promote re-establishment of previously lost synaptic connections after pre-exposure to stress.

### Ketamine increases activity of PV interneurons after RS ex vivo and in vivo

**Fig. 3:**
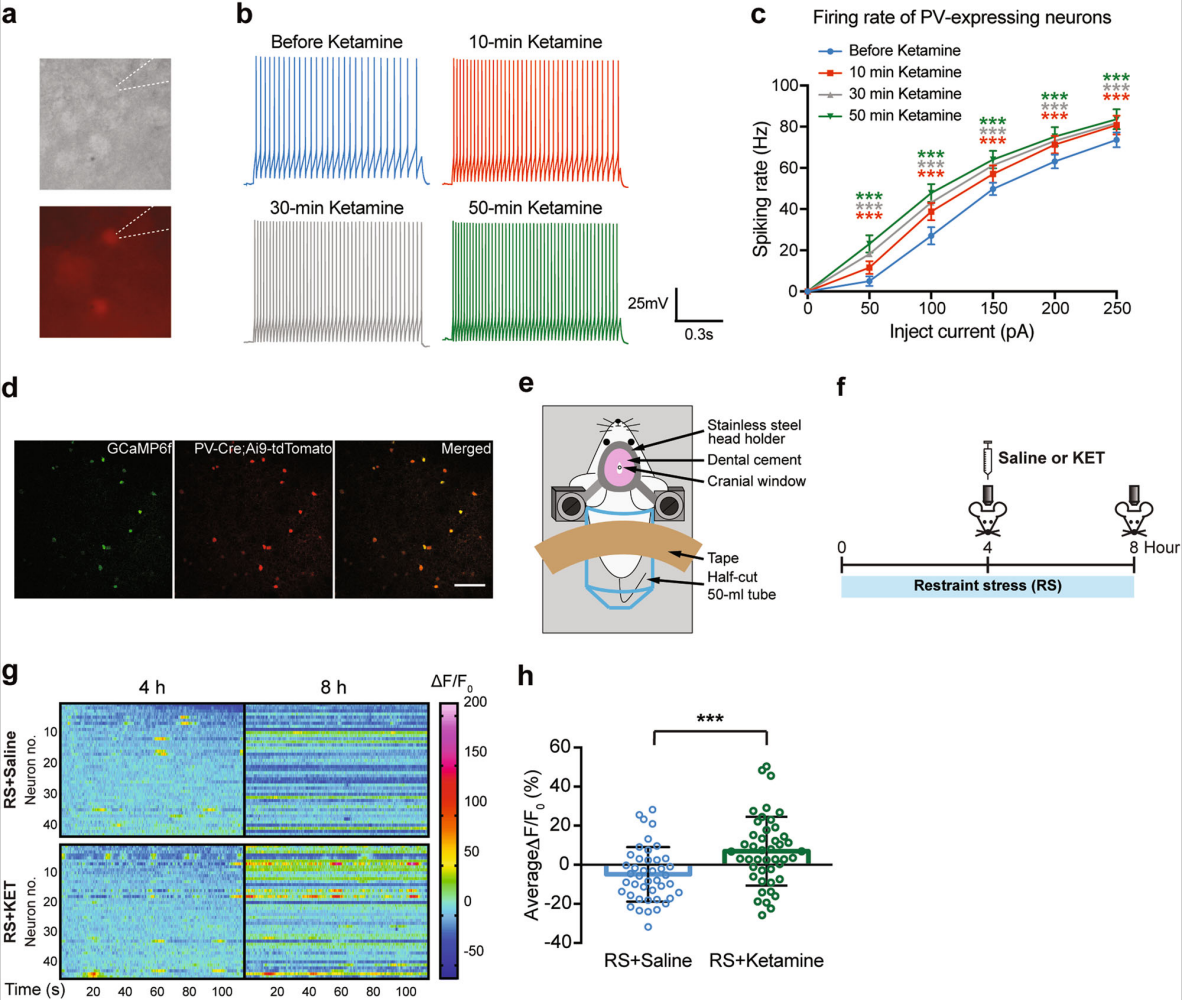
Ketamine increases PV interneurons activity ex vivo and in vivo after restraint stress. **a** Bright field and fluorescent image of PV interneurons expressing tdTomato in frontal cortical slice from restraint stressed *PV*-Cre;Ai9-tdTomato mice under whole-cell recording. Expression of tdTomato is specific to PV interneurons in *PV*-Cre;Ai9-tdTomato mice. **b** Representative traces showing evoked action potential (APs) firing of PV interneurons (100 pA) before, and 10-min, 30-min, 50-min after bath application of ketamine (10 µM). **c** Graph showing frequency of APs evoked by a range of injection currents of PV interneurons before and after ketamine. *P* < 0.0001, *F*_ketamine treatment time(3, 48)_ = 13.92, two-way repeated measure ANOVA. ***P < 0.0001 compared with before ketamine, post hoc Dunnett’s test. *n* = 17 neurons. Data are presented in mean ± s.e.m. **d** Representative in vivo two-photon image of the specific expression of Cre-dependent GCaMP6f in PV interneurons in L2/3 FrA of *PV*-Cre;Ai9-tdTomato mice. Green: GCaMP6f. Red: tdTomato. Scale bar: 60 µm. **e** Schematic diagram of Ca^2+^ imaging under RS in vivo. **f** Timeline for GCaMP6f imaging. GCaMP6f signal was collected at 4 h and 8 h. Saline or ketamine was injected right after the imaging session at 4 h. **g** Heat maps showing ΔF/F_0_ of individual neurons over the 2-min imaging session in RS + saline (*n* = 43 cells from seven mice) and RS + KET (*n* = 45 cells from nine mice) groups using 4 h reading as baseline. **h** Average ΔF/F_0_ in RS + saline and RS + KET groups after ketamine or saline treatment compared with 4 h activity. ***P < 0.001 compared with RS + saline, Student’s *t* test. Data are presented in mean ± s.d

It is proposed that ketamine preferentially suppresses inhibitory interneurons to cause disinhibition of pyramidal neurons and subsequent enhancement of excitatory transmission^32,33,59^. However, the effect of ketamine at antidepressant dose on specific interneuron subtypes in stressed animals is unclear. As PV interneuron is the major subtype of interneurons in the cortex, we examined the effect of low-dose ketamine on PV interneurons in slices containing FrA from mice prior exposed to 7 days of RS. Activity of PV interneurons was studied by patch-clamp electrophysiology (Fig. 3a). Ketamine (10 μM) significantly increased the frequency of evoked action potential of PV interneurons in the FrA from 10 minutes after ketamine application and persisted to up to 50 mins (Fig. 3b, c, *P* < 0.0001 for 50, 100, 150, 200, and 250 pA injection current, before ketamine vs. 10, 30, or 50 min after ketamine). This result suggests low-dose ketamine increases the excitability of PV interneurons after stress exposure.

Next, we performed functional calcium imaging to see if the elevation in activity of PV interneurons induced by ketamine can be observed in vivo under stress. A genetically encoded calcium indicator, GCaMP6f, was specifically expressed in PV interneurons by Cre-dependent AAV expression in *PV*-Cre;Ai9-tdTomato mice to evaluate the activity of PV interneurons (Fig. 3d). PV interneuron activity was monitored in awake, restrained mice (Fig. 3e). Calcium activities represented by GCaMP6f signal from PV interneurons in L2/3 of the FrA were recorded at 4 and 8 h of RS. Saline or ketamine was administrated after image acquisition at 4 h (Fig. 3f). Ketamine significantly increased the activity of PV interneurons at 8 h compared with saline, as shown by individual neuron activity and the average change in activity (Δ*F*/*F*_0_) (*P* = 0.0008, Fig. 3g, h). Together, these data suggest that ketamine enhances the activity of PV interneurons in the FrA under acute RS.

### Ketamine counteracts the loss of PV axonal boutons induced by repeated stress

**Fig. 4:**
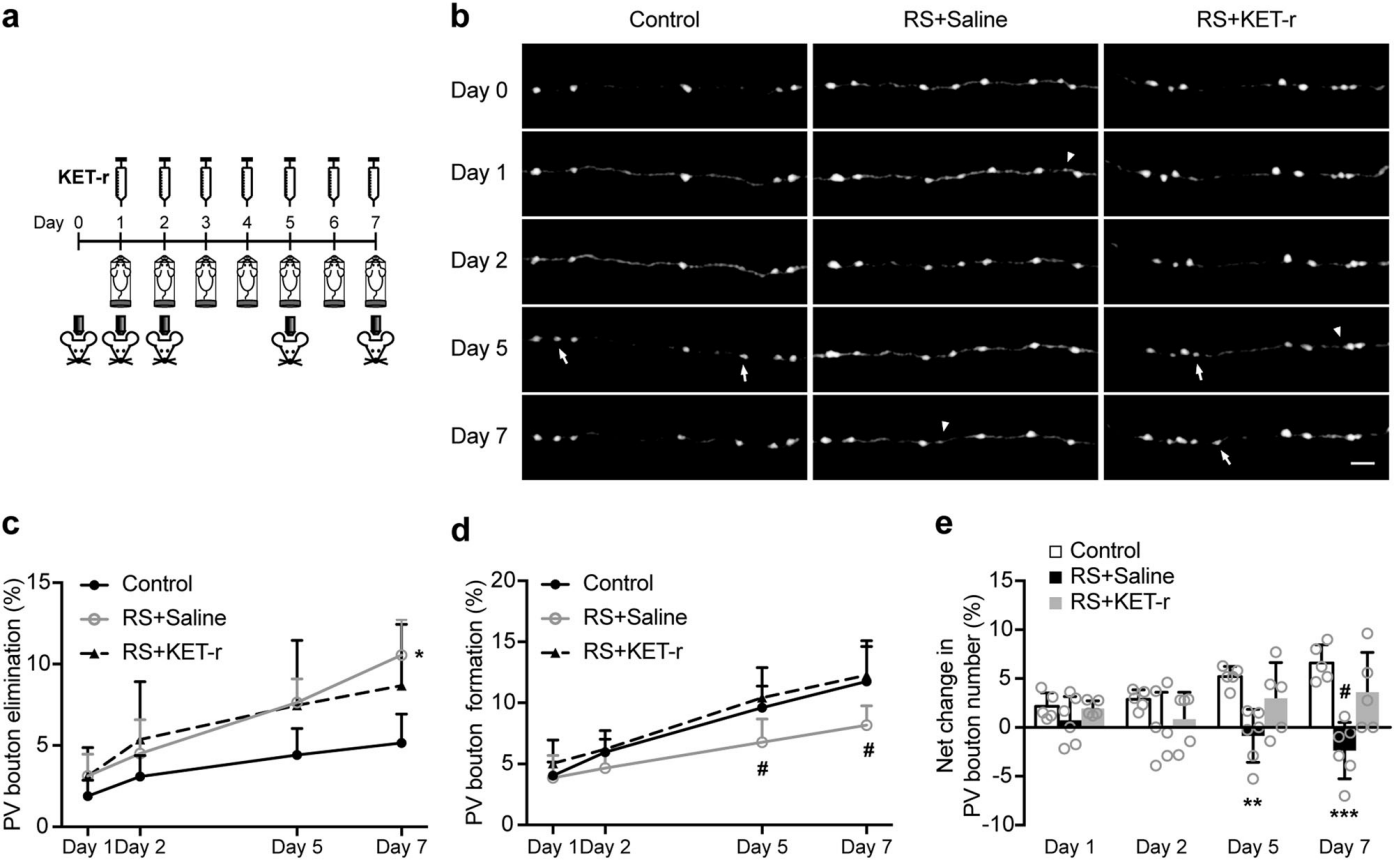
Ketamine counteracts the loss of PV axonal boutons induced by stress. **a** Timeline of PV axonal bouton imaging. Ketamine or saline was administered daily before RS sessions and mice were imaged after RS sessions. **b** Representative image of PV interneuron axonal branches in the FrA from control (*n* = 5, 1131 boutons), RS + saline (*n* = 6, 1429 boutons) and RS + KET-r (*n* = 5, 953 boutons) groups. Arrowheads mark sites of bouton elimination. White arrows mark sites of newly formed boutons. Scale bar 4 µm. **c** Rate of PV boutons elimination in control, RS + saline and RS + KET-r groups. Day 7: *P* = 0.0211, *H* = 7.05, Kruskal–Wallis test. **d** Rate of PV bouton formation in control, RS + saline and RS + KET-r groups. Day 5, *P* = 0.0264, *F*_(2, 13)_ = 4.869, one-way ANOVA. Day 7, *P* = 0.0292, *F*_(2, 13)_ = 4.696, one-way ANOVA. **e** Net change in PV bouton number in control, RS + saline and RS + KET-r groups. Day 5, *P* = 0.0080, *F*_(2, 13)_ = 7.15, one-way ANOVA. Day 7: *P* = 0.0010, *F*_(2, 13)_ = 12.35, one-way ANOVA. *P < 0.05, **P < 0.01, ***P < 0.001 compared with control, #*P* < 0.05 compared with RS + KET-r. Data are presented in mean ± s.d

To further understand the long-term effect of ketamine on PV interneurons, we next investigated how ketamine affect the structural dynamics of L2/3 PV interneuron axonal boutons in vivo under repeated RS (Fig. 4a, b). We first examined the effect of RS on the axonal boutons of PV interneurons in the FrA. We found that RS significantly enhanced elimination (Day 7: *P* = 0.0242) and notably inhibited formation of PV axonal boutons, which led to a significant net loss of PV axonal boutons from 5 days of RS onwards compared with naive control (Day 5: *P* = 0.0069; day 7: *P* = 0.0009, Fig. 4c–e). Next, we examined if KET-r would counteract the stress-induced loss of PV axonal boutons. We found that ketamine treatment had no effect on stress-induced elimination of PV axonal boutons (Fig. 4c). Instead, ketamine significantly increased the formation rate of PV axonal boutons at day 5 and day 7 of RS (Day 5: *P* = 0.0292; day 7: *P* = 0.0393, Fig. 4d), which resulted in a significantly higher number of PV boutons at day 7 than the saline group (*P* = 0.0173, Fig. 4e). These data show that repeated RS causes a net loss of PV boutons, which is counteracted by ketamine through enhancing the formation of PV axonal boutons.

### Activation of PV interneurons by chemogenetic manipulation mimics the effects of ketamine on dendritic spine plasticity

**Fig. 5:**
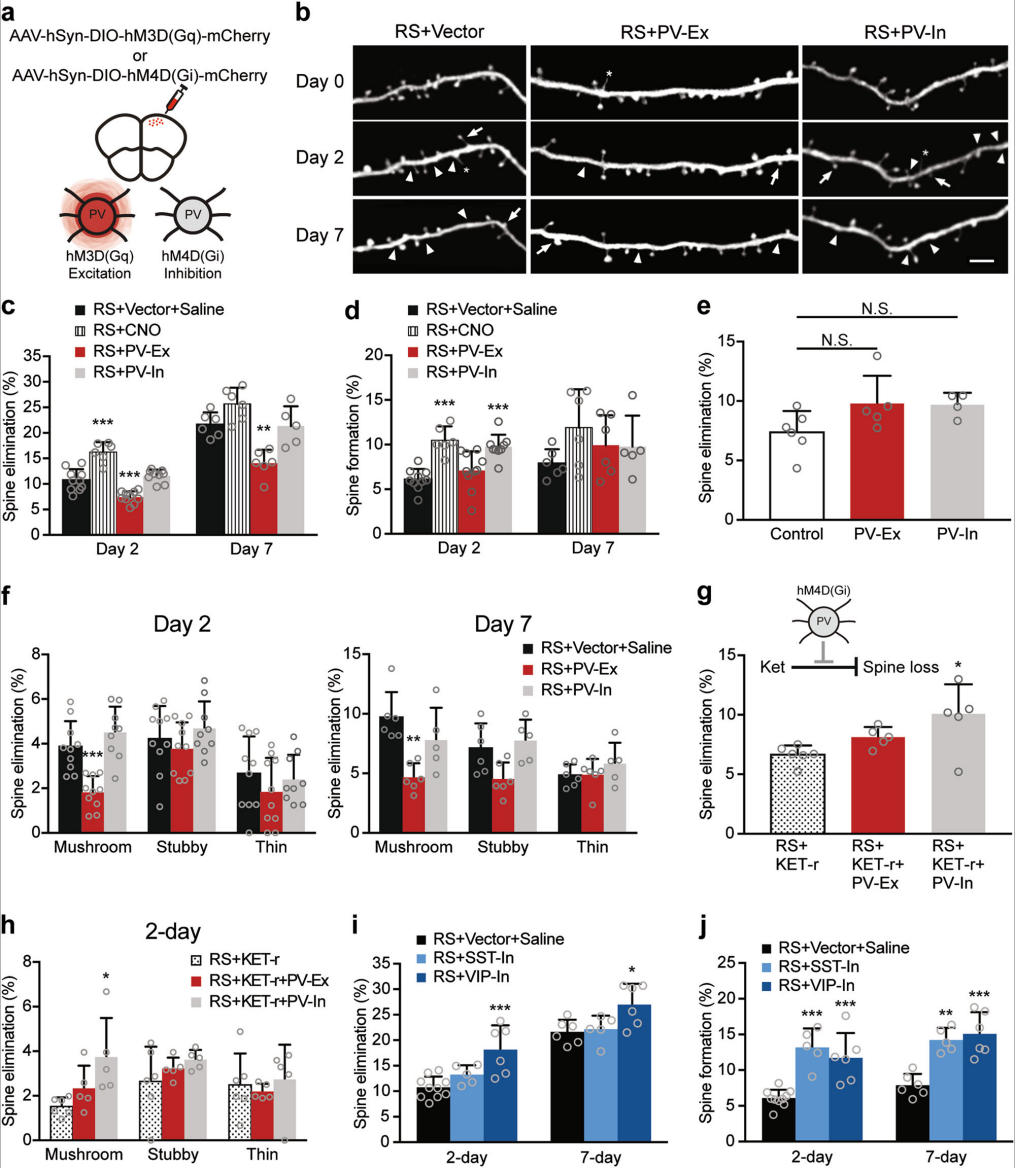
Chemogenetic activation of parvalbumin (PV)-interneurons mimics ketamine’s effect on dendritic spine plasticity under stress. **a** Schematic diagram showing the site of DREADD vectors injection. Upon CNO administration, hM3D(Gq) and hM4D(Gi) are activated to induce excitation and inhibition of PV interneurons, respectively. **b** Representative image of dendritic branches in the FrA of vector control, excitation of PV interneurons (PV-Ex) and inhibition of PV interneurons (PV-In) groups under RS. Arrowheads mark sites of spine elimination. Arrows mark sites of newly formed spines. Asterisks mark filopodia. Scale bar 4 µm. **c** Rate of dendritic spine elimination at day 2 and day 7 in RS + vector + saline, RS + CNO, RS + PV-Ex, and RS + PV-In groups. Day 2: *P* < 0.0001, *F*_(3, 31)_ = 37.56, one-way ANOVA. Day 7: *P* < 0.0001, *F*_(3, 19)_ = 15.38, one-way ANOVA. **d**) Rate of dendritic spine formation at day 2 and day 7 in RS + vector + saline, RS + CNO, RS + PV-Ex, and RS + PV-In groups. Day 2: *P* < 0.0001, *F*_(3, 31)_ = 13.94, one-way ANOVA. Day 7: *P* = 0.2576, *F*_(3, 19)_ = 1.458, one-way ANOVA. RS + Vector + saline (Day 2: *n* = 10, 1439 spines; day 7: *n* = 6, 768 spines), RS + CNO (*n* = 6, 946 spines for both day 2 and 7), RS + PV-Ex groups (Day 2: *n* = 10, 1384 spines; day 7: *n* = 6, 837 spines) and RS + PV-In (Day 2: *n* = 9, 1264 spines; day 7: *n* = 5, 743 spines). **e**) Rate of spine elimination at day 2 in non-stress control (*n* = 6, 929 spines), PV-Ex (*n* = 5, 615 spines) and PV-In (*n* = 4, 472 spines) groups. *P* = 0.0904, *F*_(2,12)_ = 2.957, one-way ANOVA. **f** Percentage of eliminated spines classified as mushroom, stubby and thin spines after 2- and 7- day RS. Mushroom spines: Day 2, *P* < 0.0001, *F*_(2,26)_ = 18.23, one-way ANOVA. Day 7, *P* = 0.0007, *H* = 10.9, Kruskal–Wallis test. **g** Rate of dendritic spine elimination at day 2 in RS + KET-r (*n* = 6, 892 spines), RS + KET-r + PV-Ex (*n* = 5, 777 spines) and RS + KET-r + PV-In (*n* = 5, 638 spines) groups. Elimination: *P* = 0.0178, *F*_(2,13)_ = 5.579, one-way ANOVA. **h** Percentage of eliminated spines classified as mushroom, stubby, and thin spines after 2 days of RS in RS + KET-r, RS + KET-r + PV-Ex, and RS + KET-r + PV-In groups. Mushroom spines: *P* = 0.0236, *F*_(2, 13)_ = 5.064, one-way ANOVA. * *P* < 0.05 compared with RS + KET-r. **i** Rate of dendritic spine elimination at day 2 and day 7 in RS + vector + saline, RS + SST-In, RS + VIP-In groups. Day 2: *P* = 0.0008, *F*_(2, 18)_ = 11.02, one-way ANOVA. Day 7: *P* = 0.0207, *F*_(2, 14)_ = 5.178, one-way ANOVA. **j** Rate of dendritic spine formation at day 2 and day 7 in RS + vector + saline, RS + SST-In, RS + VIP-In groups. Day 2: *P* < 0.0001, *F*_(2, 18)_ = 18.91, one-way ANOVA. Day 7: *P* = 0.0001, *F*_(2, 14)_ = 17.98, one-way ANOVA. RS + SST-In (Day 2: *n* = 5696 spines; day 7: *n* = 5, 676 spines), RS + VIP-In (Day 2: *n* = 6, 789 spines; day 7: *n* = 6, 744 spines). **c**, **d**, **f**, **i**, **j** *P < 0.05, **P < 0.01, ***P < 0.001 compared with RS + vector + saline. Data are presented in mean ± s.d

Based on the effects of ketamine on the activity and structural dynamics of PV interneurons, we next investigated if exciting PV interneurons alone could protect dendritic spines from stress. A chemogenetic approach, Designer Receptor Exclusively Activated by Designer Drugs (DREADD) was used to manipulate the activity of PV interneurons in the FrA^60^. L2/3 PV interneuron activity was selectively excited or inhibited by expressing Cre-dependent hM3D(Gq) or hM4D(Gi) in the FrA of *PV*-Cre; *Thy1*-YFP mice upon CNO injection^60^ (Fig. 5a, b, Supplementary Figure 4). Mice were given CNO (10 mg/kg) before each restraint sessions and imaged on day 0, 2, and 7 of RS (Fig. 5c). This dosage of CNO was used based on the previous study that showed selective inhibition of PV interneurons by DREADD promoted depressive-like behavior^61^. In view of the recent report showing CNO uptake might lead to non-DREADD-induced effect caused by CNO metabolite clozapine^62^, two groups of mice were used as control groups, including RS + vector + saline with control AAV injection and RS + CNO without AAV injection. After 2 days of RS, excitation of PV interneurons significantly suppressed spine elimination (*P* = 0.0004, Fig. 5c). Excitation of PV interneurons persistently protected dendritic spines from elimination after 7-day RS, similar to the effects of repeated ketamine treatment (*P* = 0.0018, Fig. 5c). Notably, excitation of PV interneurons alone in non-stressed mice produced no significant change in spine turnover (Fig. 5e). On the other hand, inhibition of PV interneurons did not reduce the rate of spine elimination in RS mice at both day 2 and day 7 (Fig. 5c). Instead, inhibiting PV interneurons significantly increased spine formation at day 2 when compared with vector control (*P* = 0.0001), but the effect did not persist to day 7 (Fig. 5d). CNO treatment alone did not alleviate stress-induced spine elimination and caused only a transient increase in spine formation rate in RS mice (*P* < 0.0001, Fig. 5c, d). Morphological analysis of eliminated spines showed that significantly less mushroom spines were eliminated after RS in the PV excitation group when compared with vector control (Day 2, *P* = 0.0004; Day 7, *P* = 0.0034, Fig. 5f). To understand if the effect of ketamine on dendritic spines is dependent on PV interneuron activity, mice were subjected to ketamine treatment together with PV excitation or PV inhibition. Inhibition of PV interneurons on top of ketamine treatment abolished the effects of ketamine, resulting in a significantly higher spine elimination rate (*P* = 0.0138) and a higher percentage of mushroom spines being eliminated than ketamine alone after 2-day RS (*P* = 0.0190); whereas a combination of ketamine and PV interneuron excitation had a similar effect on spine elimination as ketamine alone (Fig. 5g, h). Moreover, we investigated if the observed effect of PV excitation on dendritic spines has behavioral significance. We found that both acute ketamine and PV excitation significantly increased the percentage of open arm entries in 14-day stressed mice compared to 14-day RS + saline control in the elevated plus maze test, showing ketamine and PV excitation reversed anxiety-like behavior (Supplementary Figure 5). This result suggests that PV excitation might be involved in the behavioral effect of ketamine, in addition to the effect on dendritic spine dynamics.

To further examine if inhibition of other subtypes of interneurons induce disinhibition of PV interneurons and produce similar effects as PV interneuron excitation, we selectively inhibited the activity of SST- and VIP-expressing interneurons by expressing Cre-dependent hM4D(Gi) in *SST*-Cre; *Thy1*-YFP or *VIP*-Cre; *Thy1*-YFP mice. Our data showed that inhibition of SST or VIP interneurons did not prevent stress-induced elimination. Instead, inhibition of VIP interneurons exacerbated spine elimination persistently under RS compared with vector control (Fig. 5i, day 2: *P* = 0.0005; day 7: *P* = 0.0270). On the other hand, inhibition of either SST or VIP interneurons significantly increased spine formation rate after both 2- and 7-day of RS (SST: day 2: *P* = 0.0001; day 7: *P* = 0.0010, VIP: day 2: *P* = 0.0006 and day 7: *P* = 0.0002, Fig. 5j). Taken together, our data show that the effect of ketamine on dendritic spine plasticity in RS mice is specifically mimicked by excitation of PV interneurons but abolished by PV interneuron inhibition, suggesting that the protective effect of ketamine on spine elimination against stress involves the activation of PV interneuron.

## Discussion

Chronic stress exposure has been reported to induce dendritic spine loss and reduce dendritic arborization in prefrontal cortex and hippocampus^20,58^. Previous studies reported that ketamine increased dendritic spine density in naive or stressed animals using fixed brain tissues^26,27,29,63–65^. However, it is unclear whether the increase in dendritic spine density is owing to the promotion of spinogensis or inhibition of spine elimination. By using in vivo two-photon imaging, we traced how repeated stress affected synaptic structure dynamics in the FrA and how ketamine counteracted stress effects in 1-month-old mice. Here we found that stress-induced loss of dendritic spines was the result of reduced spine formation and enhanced spine elimination. A recent study found that repeated stress enhanced the elimination but had no effect on the formation of dendritic spines in the barrel cortex^47^. This discrepancy suggests that stress may affect dendritic spine dynamics in a region-specific manner, which could be accounted by the different connectivity^66^ and basal dendritic spine dynamics in frontal and barrel cortices^67^.

Ketamine is suggested to exert antidepressant effect through the promotion of synaptogenesis^58^, based on the previous findings that it increased dendritic spine density in naive rodents^26,28,29^ and reversed the reduction of dendritic spine density in stressed rodents^27,64,68^. Here, we found that ketamine counteracted the loss of dendritic spines by preventing stress-induced spine elimination while having minimal effect on spine formation. This discrepancy could be owing to the fact that previous reports used fixed tissue samples^26–29,64,68^ that can only compare the difference in spine number or density across different treatment groups, whereas we examined the dynamics of dendritic spines in the same animal over time in vivo. A previous report observed that ketamine increased spine formation rate in PFC of naive mice^30^. Similarly, we also found that ketamine transiently increased spine formation rate in non-stressed mice, suggesting ketamine may exert differential effect in stressed and non-stressed animal. Indeed, a recent human study reported that ketamine had distinct electrophysiological and behavioral effects in depressed and healthy subjects^69^. In addition, we showed single dose of ketamine only protected dendritic spines against repeated stress transiently, whereas repeated doses prolonged this protective effect. Our data are consistent with clinical studies that repeated doses of ketamine prolonged its antidepressant action^25,70–72^. Furthermore, ketamine significantly reduced the elimination of mushroom spines that are relatively more persistent and mature compared with other dendritic spine types^2^. Previous studies also showed ketamine increased the proportion of spines with large heads that are putative mushroom spines^27,29^. Ketamine also promoted the formation of spines in close proximity to the previously stress-eliminated spines. Owing to the heightened synaptic plasticity in adolescent mice used in the present study^48,49^, the re-formation of spines might occur at the intrinsic hot spots on dendrites^73,74^. The enhancement of such re-formation of spines by ketamine may increase the likelihood of the re-establishment of previously lost synaptic connections.

The disinhibition hypothesis proposes that ketamine exerts antidepressant action by preferential inhibition of GABAergic interneurons to disinhibit pyramidal neurons, resulting in an enhancement of excitatory synaptic transmission^32,33^. Evidences supporting this hypothesis include the observation that NMDAR antagonist MK-801 preferentially suppressed the activity of fast-spiking interneurons and caused a delayed increase in pyramidal neurons activity ex vivo^59^. Also, ketamine at psychotic dose (30 mg/kg) decreased the activity of fast-spiking interneurons but had no effect on regular-spiking neurons in rat orbitofrontal cortex in vivo^75^. Low-dose of ketamine (1 µM) was found to reduce inhibitory inputs onto pyramidal neurons, as well as increasing pyramidal neuron excitability in hippocampus ex vivo^76^. However, here we showed that ketamine at 10 mg/kg, a dosage that elicited antidepressant action in previous studies^27,29,64^, increased the activity of genetically labeled PV interneurons in the FrA of mice under acute stress in vivo. Also, 10 µM of ketamine enhanced activity of PV interneurons in frontal cortical slices prepared from repeatedly stressed mice. It is noted that we examined the effects of ketamine on PV activity under stressed condition, whereas previous studies utilized naive animals, which might explain the observed difference. Moreover, we showed here for the first time the structural dynamics of L2/3 PV axonal boutons in the frontal cortex of adolescent mice was impaired by repeated stress. The naive control mice displayed a net increase of PV boutons over seven days, whereas 5 days of stress caused a net loss of PV axonal boutons. A previous study also found that seven days of RS reduced excitability of PV interneurons^47^, suggesting repeated stress impairs PV interneurons both structurally and functionally. Ketamine counteracted the loss of PV boutons at day 5 of RS by enhancing bouton formation. These data demonstrated that ketamine enhanced both the function and structure of PV interneurons under stressed condition.

Furthermore, data from chemogenetic experiments showed that selective activation of PV interneuron prevented stress-induced spine elimination, whereas inhibition of PV interneuron abolished the protective effect of ketamine against stress-induced spine elimination, suggesting the involvement of PV interneuron activity in the modulation of dendritic spines by ketamine. In addition, we observed that selective activation of PV interneurons mimicked, whereas selective inhibition of PV interneurons abolished, the anxiolytic effect of ketamine in the elevated plus maze test (Supplementary Figure 5), suggesting the behavioral effect of ketamine also involves PV interneuron activity. Similarly, previous findings suggest ketamine may not exert its antidepressant effect through NMDAR antagonism on or by suppression of PV interneurons in the frontal cortex. For instance, a recent report showed that specific NMDAR knock out in PV interneurons did not affect ketamine’s antidepressant effect^77^. Also, selective inhibition of PV interneurons in the frontal cortex was found to promote depressive behavior^61^. In addition, other hypotheses suggest ketamine might work through alternative mechanisms other than inhibition of inhibitory interneurons, such as blockade of spontaneous NMDAR-mediated transmission and direct inhibition of extra-synaptic NMDAR containing GluN2B on pyramidal neurons, both of which would result in protein synthesis-depending enhancement of synaptic transmission^32,33^.

In the chemogenetic experiments, L2/3 PV interneurons were manipulated. PV interneuron boutons and calcium activity were also imaged in the same layers owing to the imaging depth limit for cranial window imaging. For spine imaging, apical dendrites of L5 pyramidal neurons were studied. Although L2/3 PV interneurons mainly exhibit local inhibition^43^, they also innervate L5 pyramidal neurons and PV interneurons^36,78^. In addition, L2/3 pyramidal neurons provide inputs to L5 tufted pyramidal neurons^79,80^. Thus, activation of L2/3 PV interneurons can indirectly or directly affect the activity of L5 pyramidal neurons. Although the mechanism underlying the activation of PV interneurons to counteract stress-induced spine loss remains unclear, it is possible that the prolonged increase in inhibitory inputs could induce homeostatic synaptic plasticity, which in turn would cause a compensatory enhancement of excitatory synapses^47,81,82^. A recent study also showed selective activation of PV interneurons in the barrel cortex prevented stress-induced dendritic spines loss and behavioral deficits in a texture discrimination task^47^. The mechanism of how ketamine activates PV interneurons is largely unknown. In this study, we showed that selective SST or VIP inhibition did not mimic the effect of ketamine or PV activation on spine dynamics, suggesting ketamine did not disinhibit PV interneurons by suppressing SST or VIP interneurons. Previous reports showed that ketamine-induced rapid glutamate surge^83,84^, which could in turn increase glutamatergic inputs onto PV interneurons for PV interneuron activation. Future study is needed to further investigate the mechanism underlying ketamine-induced PV activation and its role on homeostatic dendritic spine plasticity under stressed condition.

The complex effects of stress are mediated through activation of hypothalamic–pituitary–adrenal (HPA) axis and subsequent release of stress hormones. Modulation of the HPA axis was implicated in the action of ketamine. For instance, ketamine has been reported to reduce serum corticosterone (CORT) level in naive mice^85^ and reversed the increase of CORT and adrenocorticotropic hormone (ACTH) levels in chronically stressed rat^86^. Behavioral effects of repeated CORT injection were also found to be blocked by ketamine^87–89^. Moreover, antagonist for the mineralocorticoid receptor of CORT or inhibiting ACTH prevented the CORT-induced increase in spine elimination in the barrel cortex^90^. Therefore, we cannot exclude the possibility that ketamine might exert its effect on dendritic spines through regulation of the HPA axis.

To sum up, our data demonstrated that repeated low doses of ketamine persistently protected dendritic spines from the detrimental effects of stress, which was mimicked by the selective activation of PV interneurons in vivo. We also showed for the first time that ketamine at a low dose increased the activity of PV interneurons and rescued the net loss of PV axonal boutons under stress in the FrA in adolescent mice. It is noted that our observations could be region- and age-specific. Nonetheless, our study provides new insights on the effects of ketamine on synaptic circuitry under stress and a possible mechanism to counteract stress effect through PV interneuron activation.

## Supplementary information

Suppl. material
