# Supplementary material for: Ketamine and selective activation of parvalbumin interneurons inhibit stress-induced dendritic spine elimination
Source: Transl Psychiatry. 2018 Dec 10;8:272. doi: 10.1038/s41398-018-0321-5 (PMC6288154; doi:10.1038/s41398-018-0321-5)
Supplement: Supplementary file 1 — Suppl. material [file 41398_2018_321_MOESM1_ESM.pdf]

Supplementary information

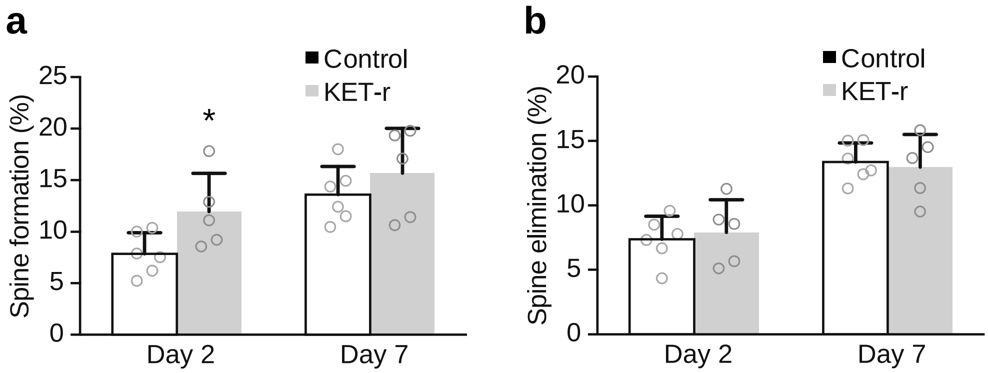

Supplementary Figure 1

Ketamine treatment has no effect on spine elimination in non-stress mice.

(**a, b**) Rate of spine formation and elimination in control (n = 6, 929 spines) and KET-r (n = 5, 588 spines) groups under non-stress condition. Repeated ketamine treatment (KET-r) increased spine formation at day 2 (P = 0.0465) but not at day 7 (P = 0.3666) compared to control, and had no effect on spine elimination. Student's t test. Data are presented in mean ± sd.

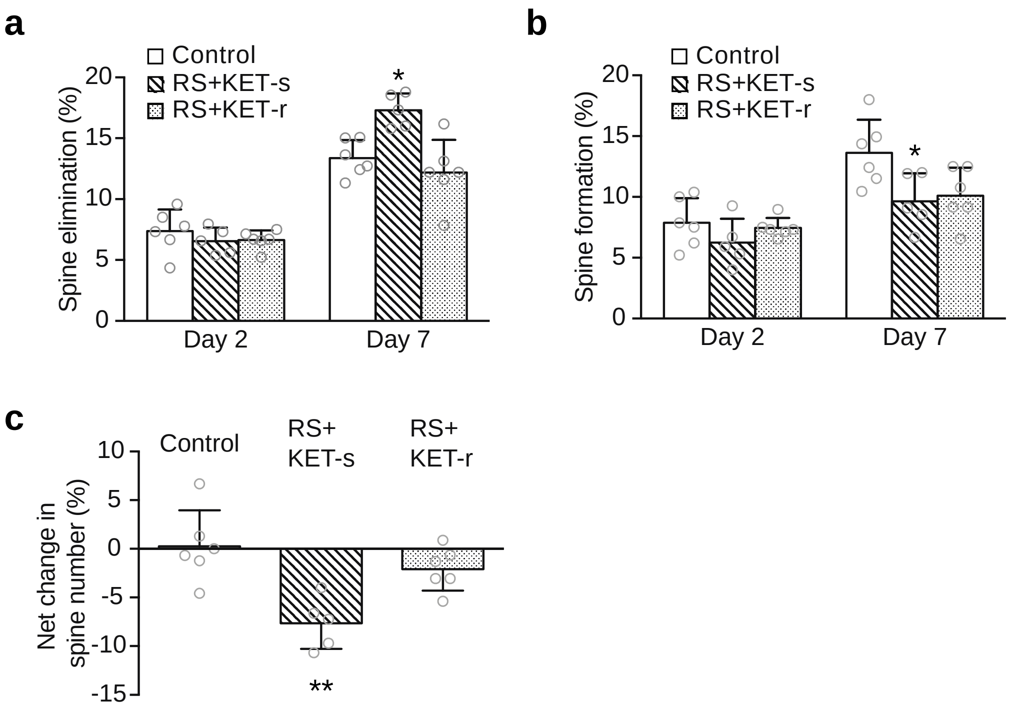

Supplementary Figure 2

Repeated ketamine treatment restores RS-induced dendritic spine loss to non-stressed control level.

(**a, b**) Rate of spine elimination and spine formation in non-stressed control, RS + KET-s, RS + KET-r groups. There was no significant difference in spine elimination and formation between control and RS + KET-r groups at both day 2 and day 7. RS + KET-s had a significantly higher elimination rate and a significantly lower formation rate compared to control at day 7 but not at day 2. (Elimination: Day 2, $P = 0.5291$, $F_{(2,14)} = 0.6664$; day 7, $P = 0.0023$, $F_{(2,14)} = 9.681$. Formation: Day 2, $P = 0.2885$, $F_{(2,14)} = 1.360$; day 7, $P < 0.0312$, $F_{(2,14)} = 4.489$.) (**c**) Net change in dendritic spine number after 7 days of RS. There was no significant difference between control and RS + KET-r groups in the net change in spine number, while RS + KET-s showed a significant loss in number of dendritic spines compared to control. $P = 0.0019$, $F_{(2,14)} = 10.19$. Control: n = 6, 929 spines. RS + KET-s: n = 5, 728 spines. RS + KET-r: n = 6, 892 spines. *$P < 0.05$, **$P < 0.01$ compared to control, one-way ANOVA followed by post-hoc Tukey's test. Data are presented in mean ± sd.

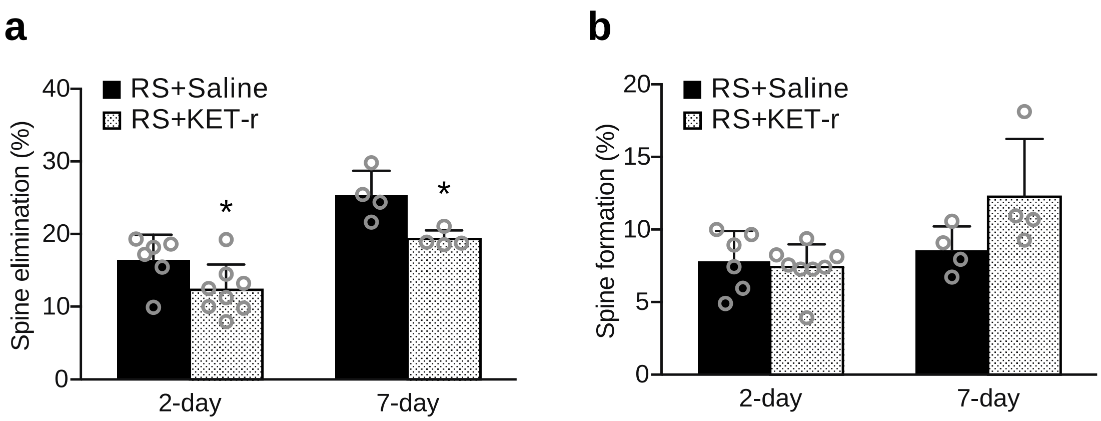

Supplementary Figure 3

Ketamine counteracts stress-induced spine elimination under isoflurane anesthesia

(**a, b**) Rate of spine elimination and spine formation in RS + Saline (Day 2: n = 6, 721 spines; day 7: n = 4, 518 spines) and RS + KET-r (Day 2: n = 8, 1107 spines; day 7: n = 4, 598 spines) groups under isoflurane anesthesia. Elimination: day 2: $P = 0.0480$, Student's *t* test; day 7: $P = 0.0286$, Mann–Whitney *U*-test. *$P < 0.05$, compared to RS + Saline. Data are presented in mean ± sd.

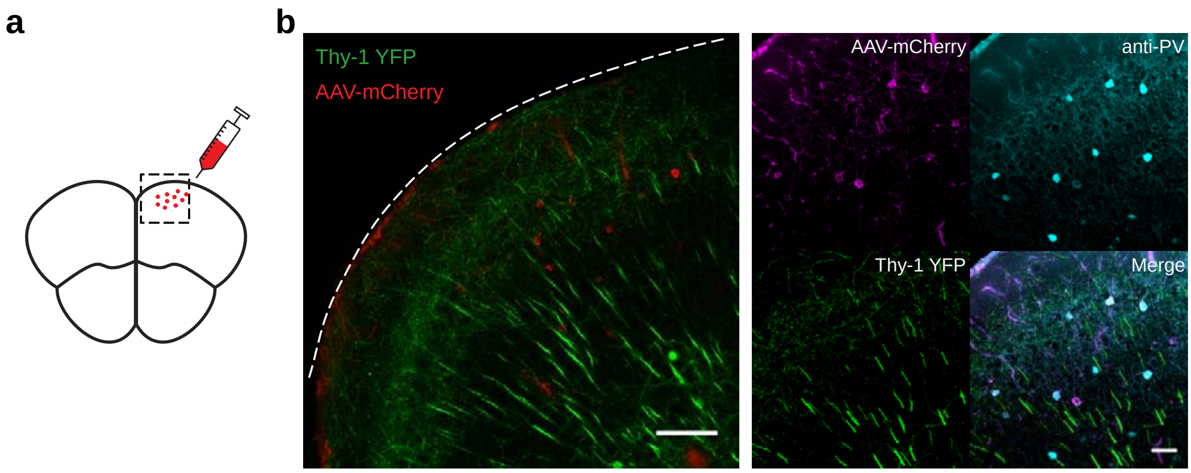

Supplementary Figure 4

Confocal images showing expression of DREADD specifically in PV interneurons in L2/3 of the FrA

(**a**) Schematic showing site of AAV injection at FrA. (**b**) Left: Representative confocal image of a coronal section taken from the region indicated by the dashed box in (a) showing DREADD-expressing neurons in the FrA. mCherry (red) indicates expression of DREADD vectors in L2/3 and YFP (green) is expressed in apical dendrites of layer 5 pyramidal neurons. Scale bar, 120 µm. Right: Images showing the selective expression of DREADD vectors (magenta) in PV interneurons (cyan). Scale bar, 50 µm.

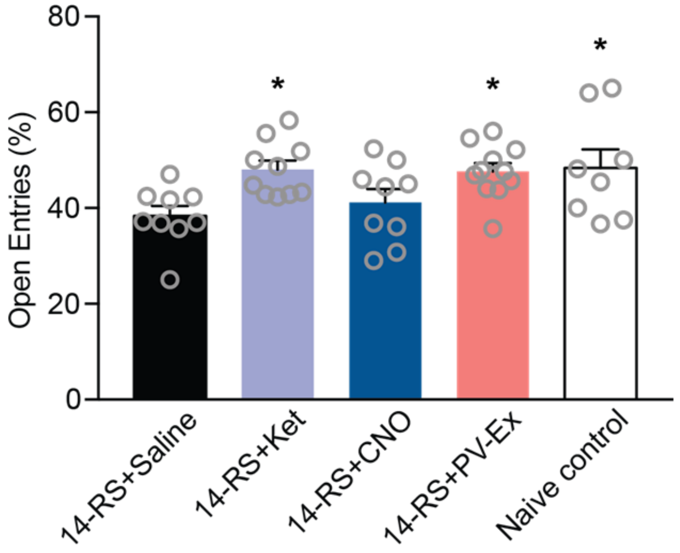

Supplementary Figure 5

PV interneurons excitation mimics ketamine's anxiolytic effect in stressed mice in EPM

Graph showing the percentage of entries into open arms in EPM in the 14-RS + Saline (n = 9), 14-RS + Ket (n = 10), 14-RS + CNO (n = 9) and 14-RS + PV-Ex (n = 11) and naïve control (n = 8) groups. 14-RS mice were restraint stressed daily for 14 days from P30, and tested on the day following the last day of stress (P44). For PV-Ex, AAV carrying vector for Cre-dependent hM3D(Gq) was injected bilaterally into FrA of *PV*-Cre/*Thy1*-YFPH mice at P20. Saline, ketamine or CNO was administered one hour before the start of EPM test. $P = 0.0140$, $F_{(4, 42)} = 3.547$, one-way ANOVA. $^*P < 0.05$ compared to 14-RS + Saline, post-hoc Dunnett's test. Data are presented in mean ± sem.

Supplementary Table 1. Analysis details for dendritic spine imaging, including the number of dendritic spine and dendritic branch analyzed, and the number and sex of animal used in different groups at different imaging intervals.

Imaging interval (days)	Group	Number of analyzed dendritic spines, dendritic branches on Day 0	Number of mice (Male, Female)
2/7	Control	929, 60	6 (3,3)
2/7	RS	910, 67	6 (3,3)
7	2d RS+5d Recovery	504,37	4 (3,1)
2/7	RS+Saline	774, 52	5 (1,4)
2/7	RS+Ket-s	728, 48	5 (2,3)
2/7	RS+Ket-r	892,65	6 (3,3)
2/7	Ket-r	588, 47	5 (3,2)
2/4	Pre-stress+Saline	735, 51	6 (1,5)
2/4	Pre-stress+Ket-r	830, 56	6 (3,3)
2	RS+Vector+Saline	1439, 94	10 (6,4)
2/7	RS+CNO	946, 58	6 (6,0)
2	RS+PV-Ex	1384, 93	10 (6,4)
2	RS+PV-In	1264, 87	9 (6,3)
2	PV-Ex	615, 53	5 (4,1)
2	PV-In	472, 36	4 (3,1)
2	RS+PV-Ex+Ket-r	777, 49	5 (0,5)
2	RS+PV-In+Ket-r	638, 50	5 (3,2)
2	RS+SST-In	696, 50	5 (3,2)
2	RS+VIP-In	789, 51	6 (6,0)
7	RS+Vector+Saline	768, 54	6 (4,2)
7	RS+PV-Ex	837, 60	6 (4,2)
7	RS+PV-In	743, 51	5 (3,2)
7	RS+SST-In	676, 49	5 (3,2)
7	RS+VIP-In	744, 50	6 (5,1)
2	(Isoflurane) RS+Saline	721, 53	6 (4,2)
2	(Isoflurane) RS+Ket-r	1107, 76	8 (7,1)
7	(Isoflurane) RS+Saline	518, 35	4 (3,1)
7	(Isoflurane) RS+Ket-r	598,39	4 (3,1)

Supplementary Table 2. Analysis details for PV bouton imaging, including the number of PV bouton analyzed and the number and sex of animals used in different groups.

Imaging interval (days)	Group	Number of analyzed PV boutons on Day 0	Number of mice (Male, Female)
1/2/5/7	Control	1131	5 (3,2)
1/2/5/7	RS+Saline	1429	6 (3,3)
1/2/5/7	RS+Ket-r	953	5 (2,3)

Supplementary Table 3. Table showing the number the number and gender of animals used in different groups in behavioral test.

Group	Number of mice (Male, Female)
RS+Saline	9 (9, 0)
RS+Ket	10 (5, 5)
RS+CNO	9 (5, 4)
RS+PV-Ex	11 (7, 4)
Naïve control	8 (4, 4)

Supplementary Table 4. Table showing details of statistical analysis of all figures.

Figure	Test	*p*-value	Post-hoc test	*p*-value (4 decimal places)
1c	Unpaired t-test	Day 2: $P = 0.0045$	NA	NA
1c	Mann–Whitney test	Day 7: $P = 0.0022$	NA	NA
1d	Unpaired t-test	Day 2: $P = 0.0179$<br>Day 7: $P = 0.0010$	NA	NA
1e	Unpaired t-test	$P = 0.0005$	NA	NA
1f	Unpaired t-test	$P = 0.0006$	NA	NA
1g	Unpaired t-test	Mushroom: $P < 0.0001$	NA	NA
1h	Mann–Whitney test	Mushroom: $P = 0.0022$	NA	NA
1g	Mann–Whitney test	Stubby: $P = 0.1797$	NA	NA
1h	Mann–Whitney test	Stubby: $P = 0.8182$	NA	NA
1g	Unpaired t-test	Thin: $P = 0.6754$	NA	NA
1h	Unpaired t-test	Thin: $P = 0.5797$	NA	NA
1i	Kruskal-Wallis test	Elimination:<br>$P = 0.0004$, $H = 10.88$	Dunn's test	Control vs. RS:<br>$P = 0.0229$<br>Control vs. 2d RS + 5d Recovery: $P = 0.0102$
1i	One-way ANOVA	Formation:<br>$P < 0.0001$, $F_{(2, 13)} = 23.39$	Tukey's test	Control vs. RS:<br>$P = 0.0006$<br>Control vs. 2d RS + 5d Recovery: $P < 0.0001$
2c	One-way ANOVA	Day 2:<br>$P = 0.0908$, $F_{(2, 13)} = 2.901$<br>Day 7:<br>$P = 0.3535$, $F_{(2, 13)} = 1.128$	NA	NA
2d	One-way ANOVA	Day 2:<br>$P < 0.0001$, $F_{(2,13)} = 38.78$<br>Day 7:<br>$P < 0.0001$, $F_{(2,13)} = 22.31$	Tukey's test	Day 2:<br>RS + Saline vs. RS + KET-s:<br>$P < 0.0001$<br>RS + Saline vs. RS + KET-s:<br>$P < 0.0001$

				Day 7:<br>RS + Saline vs. RS + KET-s:<br>$P = 0.1410$<br>RS + Saline vs. RS + KET-s:<br>$P < 0.0001$
2e	One-way ANOVA	$P < 0.0001$, $F_{(2,13)} = 21.22$	Tukey's test	RS + Saline vs. RS + KET-s:<br>$P = 0.0480$<br>RS + Saline vs. RS + KET-s:<br>$P < 0.0001$
2f	One-way ANOVA	$P = 0.5496$, $F_{(2,13)} = 0.627$	NA	NA
2g	One-way ANOVA	Day 2:<br>Mushroom:<br>$P < 0.0001$, $F_{(2,13)} = 87.99$<br>Stubby:<br>$P = 0.0651$, $F_{(2,13)} = 3.396$<br>Thin:<br>$P = 0.6504$, $F_{(2,13)} = 0.4448$<br>Day 7:<br>Stubby:<br>$P = 0.2048$, $F_{(2,13)} = 1.796$<br>Thin:<br>$P = 0.9637$, $F_{(2,13)} = 0.0371$	Tukey's test	Mushroom:<br>Day 2:<br>RS + Saline vs. RS + KET-s:<br>$P < 0.0001$<br>RS + Saline vs. RS + KET-r:<br>$P < 0.0001$
2g	Kruskal-Wallis test	Day 7:<br>Mushroom:<br>$P < 0.0001$, $H = 12.88$	Dunn's test	Day 7:<br>Mushroom:<br>RS + Saline vs. RS + KET-s:<br>$P = 0.2525$<br>RS + Saline vs. RS + KET-r:<br>$P = 0.0010$
2j	Unpaired t-test	Elimination: $P = 0.0161$<br>Formation: $P = 0.0027$	NA	NA
2k	Unpaired t-test	$P = 0.0087$	NA	NA
2m	Unpaired t-test	≤ 2 μm: $P = 0.0488$<br>2-4 μm: $P = 0.6756$<br>≥ 4 μm: $P = 0.3469$	NA	NA
3c	2-way repeated measure ANOVA	Treatment time:<br>$P < 0.0001$, $F_{(3, 48)} = 13.92$<br>Injection current:<br>$P < 0.0001$<br>Interaction:<br>$P < 0.0001$	Dunnett's test	Before ketamine vs. 10, 30 or 50 min after ketamine at 50, 100, 150, 200, 150 injection current (pA): $P < 0.0001$
3h	Unpaired	$P = 0.0008$	NA	NA

	t-test			
4c	Kruskal-Wallis test	Day 7:<br>$P = 0.0211$, $H = 7.05$	Dunn's test	Control vs. RS + Saline:<br>$P = 0.0242$
4d	One-way ANOVA	Day 5:<br>$P = 0.0264$, $F_{(2, 13)} = 4.869$<br>Day 7:<br>$P = 0.0292$, $F_{(2, 13)} = 4.696$	Tukey's test	RS + Saline vs. RS + KET-r:<br>Day 5: $P = 0.0292$<br>Day 7: $P = 0.0393$
4e	One-way ANOVA	Day 5:<br>$P = 0.0080$, $F_{(2, 13)} = 7.15$<br>Day 7:<br>$P = 0.0010$, $F_{(2, 13)} = 12.35$	Tukey's test	Control vs. RS + Saline:<br>Day 5: $P = 0.0069$<br>Day 7: $P = 0.0009$<br>RS + Saline vs. RS + KET-r:<br>Day 7: $P = 0.0173$
5c	One-way ANOVA	Day 2:<br>$P < 0.0001$, $F_{(3, 31)} = 37.56$<br>Day 7:<br>$P < 0.0001$, $F_{(3, 19)} = 15.38$	Tukey's test	Day 2:<br>RS + Vector + Saline vs. RS + CNO<br>$P < 0.0001$<br>RS + Vector + Saline vs. RS + PV-Ex<br>$P = 0.6865$<br>RS + Vector + Saline vs. RS + PV-In<br>$P = 0.0004$<br>Day 7:<br>RS + Vector + Saline vs. RS + CNO<br>$P = 0.1284$<br>RS + Vector + Saline vs. RS + PV-Ex<br>$P = 0.9984$<br>RS + Vector + Saline vs. RS + PV-In<br>$P = 0.0018$
5d	One-way ANOVA	Day 2:<br>$P < 0.0001$, $F_{(3, 31)} = 13.94$,<br>Day 7:<br>$P = 0.2576$, $F_{(3, 19)} = 1.458$	Tukey's test	Day 2:<br>RS + Vector + Saline vs. RS + CNO<br>$P < 0.0001$<br>RS + Vector + Saline vs. RS + PV-Ex<br>$P = 0.5258$<br>RS + Vector + Saline vs. RS + PV-In<br>$P = 0.0001$
5e	One-way ANOVA	$P = 0.0904$, $F_{(2,12)} = 2.957$	NA	NA
5f	One-way ANOVA	Day 2:<br>Mushroom:<br>$P < 0.0001$, $F_{(2, 26)} = 18.23$	Tukey's test	Day 2:<br>Mushroom:<br>RS + Vector + Saline vs.

		Stubby:<br>P = 0.3295, $F_{(2, 26)}$ = 1.159<br>Thin:<br>P = 0.4458, $F_{(2, 26)}$ = 0.8336<br>Day 7:<br>Stubby:<br>P = 0.0189, $F_{(2, 14)}$ = 5.336<br>Thin:<br>P = 0.4404, $F_{(2, 14)}$ = 0.87		RS + PV-Ex<br>P = 0.0004<br>RS + Vector + Saline vs.<br>RS + PV-In<br>P = 0.3857<br>Day 7:<br>Stubby:<br>RS + Vector + Saline vs.<br>RS + PV-Ex<br>P = 0.0578<br>RS + Vector + Saline vs.<br>RS + PV-In<br>P = 0.8236
5f	Kruskal-Wallis test	Day 7:<br>Mushroom:<br>P = 0.0007, H = 10.90	Dunn's test	Day 7:<br>Mushroom:<br>RS + Vector + Saline vs.<br>RS + PV-Ex<br>P = 0.0034<br>RS + Vector + Saline vs.<br>RS + PV-In<br>P = 0.8270
5g	One-way ANOVA	P = 0.0178, $F_{(2,13)}$ = 5.579	Tukey's test	RS + KET-r vs. RS + KET-r + PV-Ex:<br>P = 0.3558<br>RS + KET-r vs. RS + Ket-r + PV-In:<br>P = 0.0138
5h	One-way ANOVA	Mushroom:<br>P = 0.0236, $F_{(2, 13)}$ = 5.064<br>Stubby:<br>P = 0.3199, $F_{(2, 13)}$ = 1.246	Tukey's test	Mushroom:<br>RS + KET-r vs. RS + KET-r + PV-Ex:<br>P = 0.4971<br>RS + KET-r vs. RS + Ket-r + PV-In:<br>P = 0.0190
5h	Kruskal-Wallis test	Thin:<br>P = 0.3463, H = 2.241	NA	NA
5i	One-way ANOVA	Day 2:<br>P = 0.0008 $F_{(2, 18)}$ = 11.02<br>Day 7:<br>P = 0.0207, $F_{(2, 14)}$ = 5.178	Tukey's test	Day 2:<br>RS + Vector + Saline vs.<br>RS + SST-In:<br>P = 0.3175<br>RS + Vector + Saline vs.<br>RS + VIP-In:<br>P = 0.0005<br><br>Day 7:<br>RS + Vector + Saline vs.<br>RS + SST-In:<br>P = 0.9629

				RS + Vector + Saline vs. RS + VIP-In: $P = 0.0270$
5j	One-way ANOVA	Day 2: $P < 0.0001$, $F_{(2, 18)} = 18.91$ Day 7: $P = 0.0001$, $F_{(2, 14)} = 17.98$	Tukey's test	Day 2: RS + Vector + Saline vs. RS + SST-In: $P = 0.0001$ RS + Vector + Saline vs. RS + VIP-In: $P = 0.0006$ Day 7: RS + Vector + Saline vs. RS + SST-In: $P = 0.0010$ RS + Vector + Saline vs. RS + VIP-In: $P = 0.0002$
Suppl. 1a	Unpaired t-test	Day 2: $P = 0.0465$ Day 7: $P = 0.3666$	NA	NA
Suppl. 1b	Unpaired t-test	Day 2: $P = 0.6867$ Day 7: $P = 0.7588$	NA	NA
Suppl. 2a	One-way ANOVA	Day 2: $P = 0.5291$, $F_{(2,14)} = 0.6664$ Day 7: $P = 0.0023$, $F_{(2,14)} = 9.681$	Tukey's test	Day 7: Control vs. RS + KET-s: $P = 0.0145$ Control vs. RS + KET-r: $P = 0.5706$
Suppl. 2b	One-way ANOVA	Day 2: $P = 0.2885$, $F_{(2,14)} = 1.360$ Day 7: $P = 0.0312$, $F_{(2,14)} = 4.489$	Tukey's test	Day 7: Control vs. RS + KET-s: $P = 0.0457$ Control vs. RS + KET-r: $P = 0.0651$
Suppl. 2c	One-way ANOVA	$P = 0.0019$, $F_{(2,14)} = 10.19$	Tukey's test	Control vs. RS + KET-s: $P = 0.0015$ Control vs. RS + KET-r: $P = 0.3778$
Suppl. 3a	Unpaired t-test	Day 2: $P = 0.0480$	NA	NA
Suppl. 3a	Mann–Whitney test	Day 7: $P = 0.0286$	NA	NA
Suppl. 3b	Mann–Whitney test	Day 2: $P = 0.5495$	NA	NA
Suppl. 3b	Unpaired t-test	Day 7: $P = 0.1389$	NA	NA
Suppl. 5	One-way ANOVA	$P = 0.0140$, $F_{(4, 42)} = 3.547$	Dunnett's test (compared to RS + Saline)	14-RS + Saline vs. 14-RS + Ket: $P = 0.0245$ 14-RS + Saline vs. 14-RS + CNO:

				P = 0.8379<br>14-RS + Saline vs.<br>14-RS + PV-Ex:<br>*P* = 0.0282<br>14-RS + Saline vs.<br>Naive control:<br>*P* = 0.0295

Supplementary Method

Elevated Plus Maze (EPM)

An EPM containing two opposing open arms and two opposing closed arms (height: 40 cm, length: 40 cm) forming a cross-shape was used. Each mouse was placed into an EPM facing an open arm and allowed to freely explore for 5 minutes. ANY-maze software (Stoelting, USA) was used for recording and analysis. The number of entries into the open and closed arms was counted.
